# Sports preferences in children and adolescents in psychiatric care—evaluation of a new questionnaire

**DOI:** 10.3389/frcha.2024.1354595

**Published:** 2024-05-09

**Authors:** Florian Breido, Sebastian Stumm, Ekkehart Jenetzky, Michael Huss

**Affiliations:** ^1^ Department of Child and Adolescent Psychiatry, Medical Center of the Johannes Gutenberg University, Mainz, Germany; ^2^ Department of Sports Psychology, Johannes Gutenberg University, Mainz, Germany; ^3^ Institute of Integrative Medicine, Witten/Herdecke University, Witten, Germany

**Keywords:** Sports Preference Questionnaire, mental disorder, sports therapy, psychological effect, physical activity

## Abstract

**Introduction:**

As part of an exploratory and hypothesis-generating study, we developed the Sports Preference Questionnaire (SPOQ) to survey the athletic behavior of mentally ill children and adolescents, subjectively assessed physical fitness and perceived psychological effects of physical activity.

**Methods:**

In a department of child and adolescent psychiatry, we classified 313 patients (6–18 years) according to their primary psychiatric diagnosis. The patients or—in the parental version of the questionnaire—their parents reported their sport preferences on the SPOQ. As possibly influential factors, we also assessed the frequency of physical activity, the importance of a trainer, coping with everyday life through physical activity, and subjectively perceived physical fitness.

**Results:**

One in 3 patients (32.4%) stated that they were not physically active. Patients diagnosed with eating disorders reported, on average, a notably high frequency (median of 3 h/week) and degree of coping with daily life through physical activity (median of 5 on a 6-point Likert scale). Patients with anxiety disorders and depression had the lowest self-perception of physical fitness (mean value of 3.1 or 3.7 on an interval scala from 0 to 9). The presence of a trainer was generally considered not important, except for ADHD patients (median of 3 on a 6-point Likert scale).

**Conclusion:**

The SPOQ is sensitive for differential effects of core child and adolescent disorders as well as for main covariates influencing the complex association between physical activity and emotional and behavioral disorders in children and adolescents. Based on this pilot study, we discussed the need for an efficacy study to measure the effects of sports therapy.

## Key practitioner message

• One in 3 patients not physically active
• High coping with daily life through physical activity in eating disorder patients
• Lowest self-perception of physical fitness in patients with anxiety disorders
• The presence of a trainer most important in ADHD patients

## 1 Introduction

The positive effect of physical activity on general health is well known and has already been proven in numerous studies. For example, reduced risk of cardiovascular disease, stroke, and cancer as well as improved stress regulation through regular exercise have been shown (1, 2).

Physical activity is also a very effective and suitable means of preventing mental illness and improving mental health [e.g., (3–6)]. When investigating the psychological effects of physical activity, positive effects on mood, psychological well-being and the concept of self and body could be demonstrated (4, 7). The effect of exercise has also been investigated at the neurobiological level. Changes in the metabolism of serotonin and dopamine in the brain have been demonstrated (8). These messenger substances play a decisive role in the development and maintenance of many mental illnesses such as depression, anxiety disorder, and attention deficit hyperactivity disorder (9–11).

Under the right conditions in organized sports, children and adolescents may develop daily life-skills such as moral reasoning, emotional control, personal responsibility, and the ability to work in teams and set goals (12). In the literature, there are a few specific sports therapeutic approaches for mentally ill children and adolescents, for example in the areas of climbing therapy, archery, and endurance training (13–16). A German study with ADHD patients showed that both long-term, natural sports therapy, and high-intensity interval training (HIT) tend to have a positive influence on the main symptoms, self-esteem and social competence (17). Nevertheless, there are only a few confirmatory studies on the content, methodology, and structure of sports therapies that do not allow generalized statements on the effects and mechanisms of action in mentally ill children and adolescents (18).

While the effects of physical activity on health have been well researched, it still seems to be largely unclear what psychological conditions need to be in place for effective sports intervention in mentally ill children and adolescents. According to Lambert (19), a healthy therapeutic relationship is a helpful factor in psychotherapy. Because a skilled coach also performs therapeutic functions in sports therapy, he could play a similar role. The desire for enjoyment in physical activity appears to be the best predictor of commitment (20). As a result, the sports treatment provided should be as appropriate as feasible, according to the individual’s demands and preferences. However, a relationship between the incidence of eating disorders and aesthetic sports such as ballet or gymnastics, but also athletics should be explored (21, 22). Psychotherapy research has shown that resource-activating strategies achieve higher therapeutic success (23). This finding could be integrated into sports therapy by focusing on the patients’ athletic abilities and personal goals. Kirkaldy et al. (5), also point out that more positive feedback regarding physical activity and social recognition leads to a better self-image. This seems to be of particular importance, as people with mental disorders often feel, that they have insufficient athletic self-efficacy (24). In the long term, sports therapy according to the above-mentioned aspects, could be a good way to achieve regular exercise and thus an improvement in physical fitness and mental health, even beyond the therapeutic intervention.

However, there are almost no scientific findings on the athletic behavior of mentally ill children or adolescents and the successful implementation of sports therapy in child and adolescent psychiatry. Therefore, the aim of the present study is to explore basic findings as a first step of evidence-based sports therapy in the child and adolescent psychiatric setting. To collect new data in our clinic, existing questionnaires on athletic behavior were considered, e.g., the ATPAD scale (25), AMS-Sport (26), and EMI-2 (27). Another very detailed questionnaire for sports interests asks about sports, sports games, and orientation (28). However, no questionnaire was found that captures physical activity preferences considering the aspects of self-perception, resource activation, and problem-solving in the context of a structured sports therapy history. Consequently, the Sport Preference Questionnaire (SPOQ) was designed. The SPOQ aims to investigate the athletic behavior of mentally ill children and adolescents, assess physical fitness and perceived psychological impacts of physical activity, and develop both general and disorder-specific hypotheses.

## 2 Material and methods

### 2.1 SPOQ questionnaire

The SPOQ was created in 2015 by the authors to investigate the out-of-school athletic behavior of children and adolescents aged six to 18. The item pool includes questions based on psychotherapy criteria such as therapeutic connection, resource activation, and problem-solving (29). We expected that physical activity with more of these features would be more useful for mental health. The item pool was constructed by a team of sports scientists, psychiatrists, and psychotherapists, taking into account the literature stated in the introduction. Two versions were designed, the parental and the self-report version (from ten years old). The data were collected with the German versions of the questionnaire. Both German versions were translated into English by a bilingual American and were translated back by a bilingual German. In the first version, thirty-nine percent of the questions regarding the importance of the relationship with the trainer and daily coping through physical activity were left unanswered. These patients were returned their unfilled SPOQ one day after submission to complete the missing data. Most patients indicated that they had overlooked the questions, which is why the layout was optimized after 2 months of data collection.

The SPOQ consists of five sections (see attachment). The first section records the frequency of physical activity in the last six months in hours per week. There is also a free field to write down another frequency. Besides, there is a statement that can be marked with a cross, indicating that the respondent has never been physically active.

The second section records up to three types of physical activity that have been regularly exercised at least in the last six months. Also, the location where the physical activity is performed is recorded. There are three answer options possible: Sports Club, Gym and Other. On a Likert scale from “Not at all” (1) to “Very much” (6), participants specify their enjoyment of this type of activity. The numbers 2–5 on the Likert scale are without description.

The third section is designed as 2 statements and asks for a currently important relationship with at least 1 trainer and the current daily coping through physical activity. Again, answers are recorded using a Likert scale from 1 to 6.

The fourth section records up to five types of activity ever tried during the participant's lifetime. Also, the duration, expressed in months, is recorded. The enjoyment of types of activities is measured as in the second section.

Section Five asks for the subjective assessment of the physical fitness of the 163 patients to measure their athletic self-efficacy. This assessment is done via an interval scale from “Not physically fit” (0) to “Very physically fit” (9). The numbers 2–8 are without description.

It takes about 5 min to complete the SPOQ. The parental and self-report versions of the SPOQ can be used for free disposal. The German versions can be requested from authors.

### 2.2 Mental disorders

The type of mental disorder was diagnosed by trained clinical psychologists or psychiatrists according to ICD-10 (30), subject to the regulations. For this purpose, semi-structured clinical interviews were used in line with Sheehan et al. (31). The diagnoses were confirmed with disorder-specific questionnaires if this was necessary to complete the symptom profiles.

### 2.3 Participation

**Figure 1:**
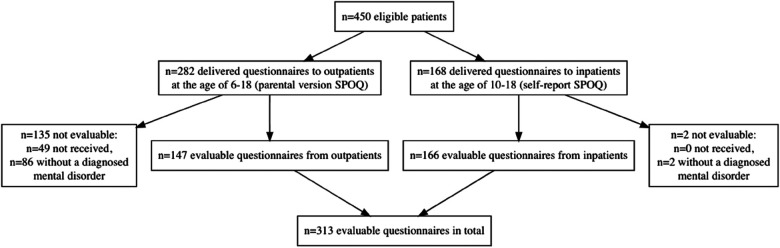
Report on patient selection.

From October 2015 to October 2017, questionnaires were send to 450 patients, of whom were registered as an inpatients or outpatients at the Child and Adolescent Psychiatry from the ages of 6.0–18.0 years. One hundred and sixty-eight inpatients received the self-report SPOQ, while 282 parents of outpatients received the parental version as part of the general registration process. It was explicitly ensured that only 1 SPOQ was available for each patient. Since the questionnaire was obligatory for inpatients, all 168 questionnaires were received back from the self-report. Two inpatients without a diagnosed disorder were excluded. One hundred and thirty-five questionnaires from the parental version were excluded because of no return or no diagnosed mental disorder. All included questionnaires covered a total of 313 patients with a mental disorder in the evaluation, 166 inpatients (self-report), and 147 outpatients (parental version). The concrete division and response rate are clearly shown in a flow chart (Figure 1).

### 2.4 Data collection and analysis

Data were collected and managed using REDCap research electronic data capture tools (32). Since the study design was strictly exploratory, we did not use inference statistics but kept analyses on a descriptive level. The statistics were calculated with R 3.6.2 (33). The flowchart to report patient selection, Figure 1, was made using the package “DiagrammeR” (34). Boxplots, line plots, and the heatmaps to show sports preferences in boys and girls were generated with the package “ggplot2” (35).

**Figure 2:**
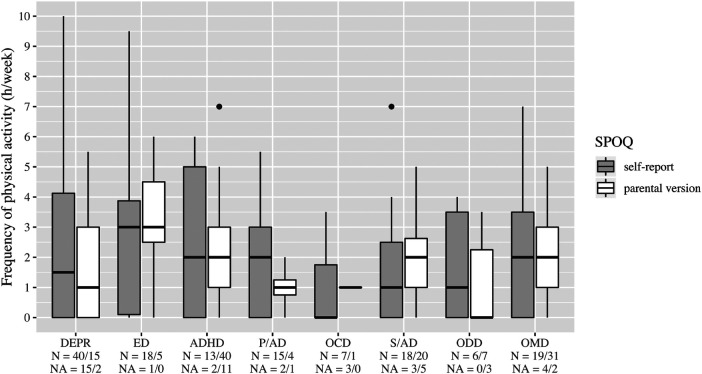
Frequency of physical activity. DEPR, depression; ED, eating disorder; ADHD, attention deficit hyperactivity disorder; P/AD, phobia/Anxiety disorder; OCD, obsessive compulsive disorder; S/AD, stress/adjustment disorder; ODD, oppositional defiant disorder; OMD, other mental disorders.

**Figure 3:**
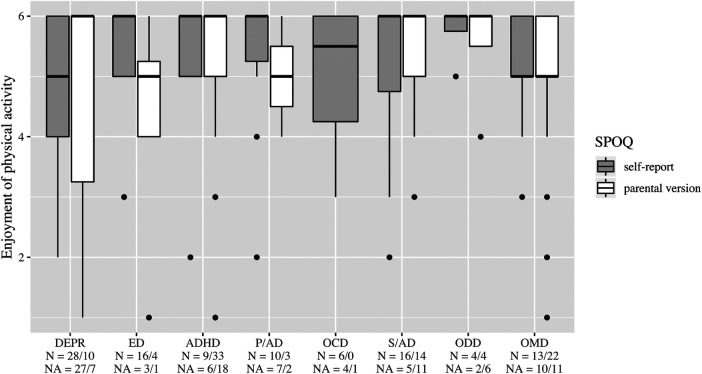
Enjoyment of physical activity (related to the first mentioned physical activity. DEPR, depression; ED, eating disorder; ADHD, attention deficit hyperactivity disorder; P/AD, phobia/anxiety disorder; OCD, obsessive compulsive disorder; S/AD, stress/adjustment disorder; ODD, oppositional defiant disorder; OMD, other mental disorders.

**Figure 4:**
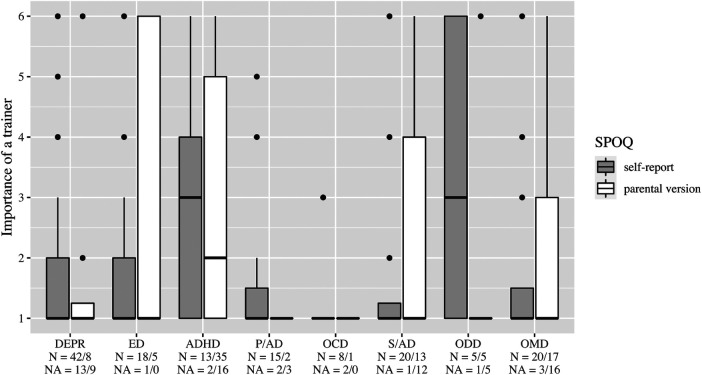
Importance of a trainer. DEPR, depression; ED, eating disorder; ADHD, attention deficit hyperactivity disorder; P/AD, phobia/anxiety disorder; OCD, obsessive compulsive disorder; S/AD, stress/adjustment disorder; ODD, oppositional defiant disorder; OMD, other mental disorders.

**Figure 5:**
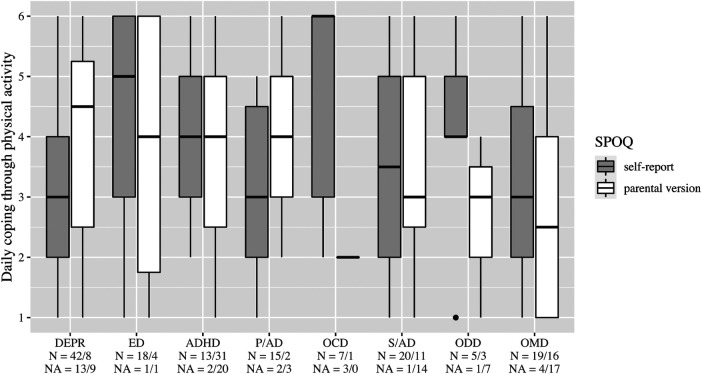
Daily coping through physical activity. DEPR, depression; ED, eating disorder; ADHD, attention deficit hyperactivity disorder; P/AD, phobia/anxiety disorder; OCD, obsessive compulsive disorder; S/AD, stress/adjustment disorder; ODD, oppositional defiant disorder; OMD, other mental disorders.

**Figure 6:**
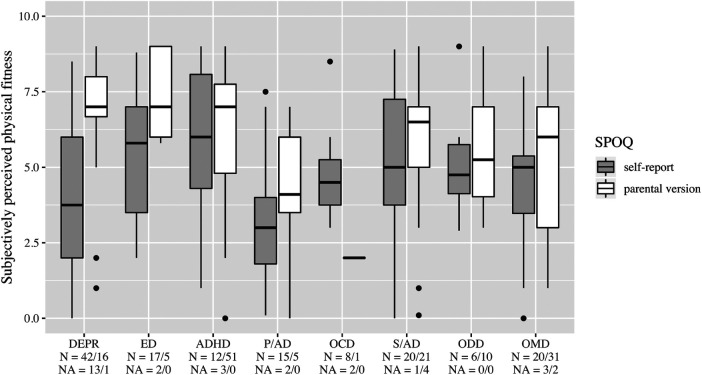
Subjectively perceived physical fitness. DEPR, depression; ED, eating disorder; ADHD, attention deficit hyperactivity disorder; P/AD, phobia/anxiety disorder; OCD, obsessive compulsive disorder; S/AD, stress/adjustment disorder; ODD, oppositional defiant disorder; OMD, other mental disorders.

**Figure 7:**
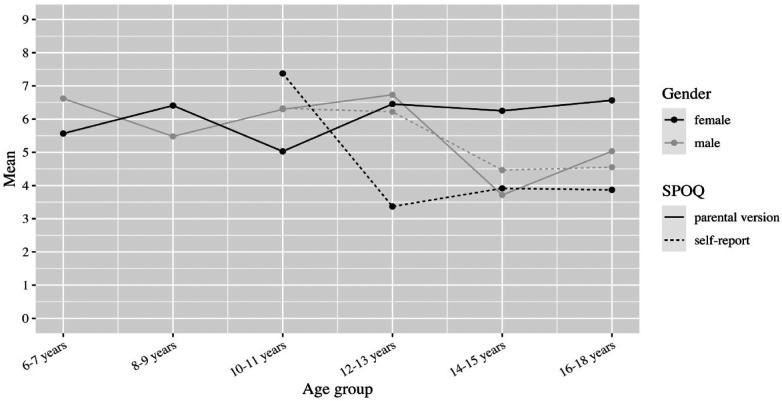
Subjectively perceived physical fitness by age and gender.

**Figure 8:**
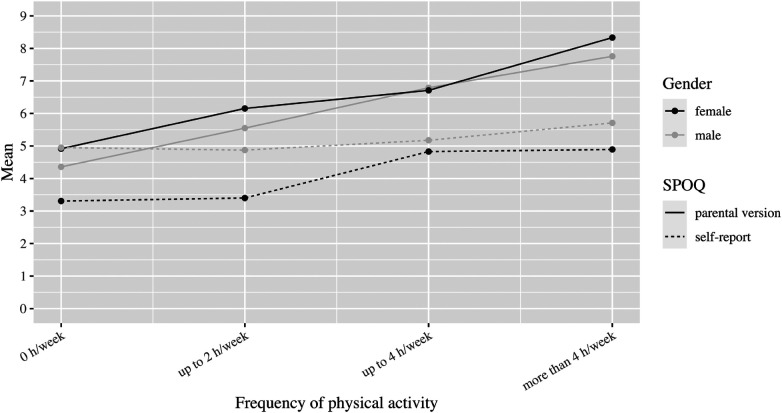
Subjectively perceived physical fitness by frequency of physical activity and gender.

**Figure 9:**
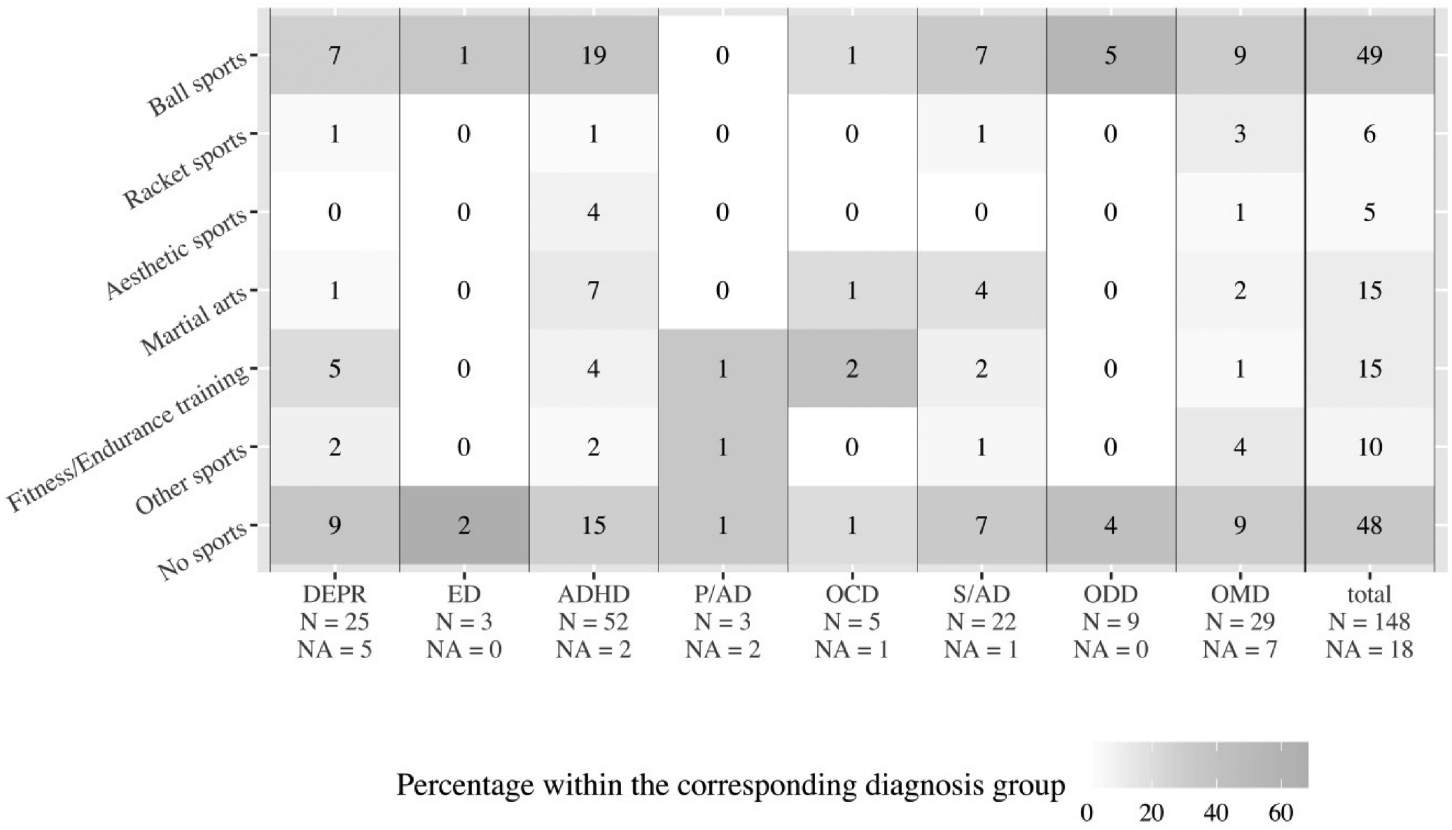
First-mentioned type of physical activity in boys. DEPR, depression; ED, eating disorder; ADHD, attention deficit hyperactivity disorder; P/AD, phobia/anxiety disorder; OCD, obsessive compulsive disorder; S/AD, stress/adjustment disorder; ODD, oppositional defiant disorder; OMD, other mental disorders; Ball sports means handball, soccer, basketball; Racket sports means table tennis, tennis, badminton; Aesthetic sports means dancing, gymnastics, vaulting; Martial arts means karate, judo, boxing; Fitness/Endurance training means gym, weight training, cycling, jogging; Other sports means swimming, climbing, athletics.

**Figure 10:**
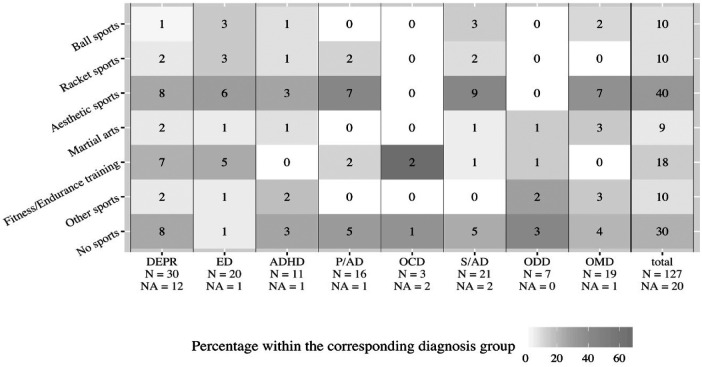
First-mentioned type of physical activity in girls. DEPR, depression; ED, eating disorder; ADHD, attention deficit hyperactivity disorder; P/AD, phobia/ anxiety disorder; OCD, obsessive compulsive disorder; S/AD, stress/adjustment disorder; ODD, oppositional defiant disorder; OMD, other mental disorders; Ball sports means handball, soccer, basketball; Racket sports means table tennis, tennis, badminton; Aesthetic sports means dancing, gymnastics, vaulting; Martial arts means karate, judo, boxing; Fitness/Endurance training means gym, weight training, cycling, jogging; Other sports means swimming, climbing, athletics.

Boxplots, Figures 2–6, were generated by diagnosis group and type of questionnaire for the frequency of physical activity, enjoyment of physical activity (related to the first-mentioned physical activity), the importance of a trainer, daily coping through physical activity, and subjectively perceived physical fitness. Therefore, N counts for the number of considered cases and NA for the number of missing values. Two line plots, Figures 7, 8, were generated to show the mean of subjectively perceived physical fitness by age, respectively frequency of physical activity, separated for gender and type of questionnaire. Six patients reported more than 10 h of physical activity per week in the self-report questionnaire. We assumed that corresponding patients were not able to estimate the actual time. Therefore, the following data were considered missing. To present the first-mentioned type of physical activity for girls, and boys heatmaps, Figures 9, 10, were created. Therefore, the mentioned types of physical activity were later aggregated into six branches by the authors. The weight, the percentage of the mentioned type in the corresponding diagnosis group, was shown through gray gradation. Table 1 shows the main characteristics of the data.

**Table 1:** Table of characteristics.

Diagnosis group	Total number (%)	Median age, years (range)	Male (%)/female (%)
All			
Total	313 (100)	13.8 (6.1, 18)	166 (53)/147 (47)
DEPR	72 (23)	15.1 (10, 18)	30 (42)/42 (58)
ED	24 (8)	15.6 (11.2, 18)	3 (12)/21 (88)
ADHD	66 (21)	10.4 (6.2, 16.7)	54 (82)/12 (18)
P/AD	22 (7)	15.7 (11.8, 17.8)	5 (23)/17 (77)
OCD	11 (4)	16.1 (13.2, 17.4)	6 (55)/5 (45)
S/AD	46 (15)	12.3 (6.1, 18)	23 (50)/23 (50)
ODD	16 (5)	13 (6.8, 16.6)	9 (56)/7 (44)
OMD	56 (18)	11.9 (6.1, 17.9)	36 (64)/20 (36)
Self-report			
Total	166 (100)	15.1 (10.8, 18)	69 (42)/97 (58)
DEPR	55 (33)	15 (12.2, 18)	20 (36)/35 (64)
ED	19 (11)	15.6 (13.2, 18)	1 (5)/18 (95)
ADHD	15 (9)	14 (11.5, 16.2)	15 (100)/0 (0)
P/AD	17 (10)	16.3 (11.8, 17.8)	4 (24)/13 (76)
OCD	10 (6)	15.9 (13.2, 17.4)	5 (50)/5 (50)
S/AD	21 (13)	14.9 (10.8, 17.4)	7 (33)/14 (67)
ODD	6 (4)	13.1 (10.9, 16.6)	2 (33)/4 (67)
OMD	23 (14)	14.1 (11.1, 17.9)	15 (65)/8 (35)
Parental version			
Total	147 (100)	10.6 (6.1, 18)	97 (66)/50 (34)
DEPR	17 (12)	15.2 (10, 17.4)	10 (59)/7 (41)
ED	5 (3)	15.9 (11.2, 17.8)	2 (40)/3 (60)
ADHD	51 (35)	9.2 (6.2, 16.7)	39 (76)/12 (24)
P/AD	5 (3)	14.2 (12.3, 16.8)	1 (20)/4 (80)
OCD	1 (1)	16.2 (16.2, 16.2)	1 (100)/0 (0)
S/AD	25 (17)	10.9 (6.1, 18)	16 (64)/9 (36)
ODD	10 (7)	11.2 (6.8, 15)	7 (70)/3 (30)
OMD	33 (22)	9.1 (6.1, 17.6)	21 (64)/12 (36)

An ethics vote with the number 837.515.15 (10833) from the ethics review board of the Landesärztekammer Rheinland-Pfalz exists for this study. Following the advice of the ethics review board, we did not assess individual informed consent statements since the procedure was fully integrated in our clinical routine assessment.

## 3 Results

### 3.1 Patients

A total number of 313 patients could be taken into account. They could be grouped into eight diagnostic categories according to the predominant mental disorder: Depression (DEPR) *N* = 72, median age = 15.1, eating disorders (ED) *N* = 24, median age = 15.6, attention deficit hyperactivity disorder (ADHD) *N* = 66, median age = 10.4, phobia/anxiety disorders (P/AD) *n* = 22, median age = 15.7, obsessive compulsive disorder (OCD) *n* = 11, median age = 16.1, stress/adjustment disorder (S/AD) *n* = 46, median age = 12.3, oppositional defiant disorder (ODD) *n* = 16, median age = 13 and other mental disorders (OMD) *n* = 56, median age = 11.9. Diagnostic group assignment was done by the primary psychiatric diagnosis on the first axis of the multi-axial diagnostic system (ICD-10) irrespectively from comorbidities.

In the inpatient group (self-report), DEPR was the predominant disorder, accounting for 33% of cases. The median age was 15.1 years, and 58% were female. In the outpatient group (parental version), ADHD was the leading disorder with 35% of the cases. The median age was 10.6 years, and 66% were male.

The complete main characteristics, overall and subdivided by questionnaire type, can be found in Table 1.

### 3.2 Athletic behavior

On average, patients with ED stated a higher frequency of physical activity than the other diagnosis groups (Figure 2). In all diagnostic groups, high enjoyment of physical activity was reported with medians between 5 and 6, however, with a wide variety in the group of patients with DEPR (Figure 3). The importance of a trainer was rated very low with a median of 1 in almost all groups. Only patients with ADHD and ODD and parents of children with ADHD rated it higher at the median (Figure 4). In the assessment of daily coping through physical activity, there was a wide range. In the self-report, patients with ED and OCD gave the highest scores and patients with DEPR and P/AD the lowest scores, on average (Figure 5). There is a discrepancy between self-report and parental version regarding subjectively perceived physical fitness. In the external assessment, higher values were indicated, on average. Physical fitness was rated particularly low in the self-assessment of patients with DEPR and P/AD (Figure 6).

If we look at the mean values of subjectively perceived physical fitness as a function of age, the parental questionnaire for girls shows only minor deviations (mean values between 5.0 and 6.6). For boys, the mean value in the parental assessment varies similarly to the girls between 5.0 and 6.7, but with a dip at the age of 14 to 15 years (mean value 3.7). In self-perception, physical fitness at the age of 10–11 years is initially rated good on average by both genders (mean value 7.4 for girls and 6.3 for boys). However, at ages 12–13 for girls and 14–15 for boys, the mean drops sharply to 3.4 and 4.5, respectively (Figure 7). Let us now consider the dependence of subjectively perceived physical fitness on the frequency of physical activity. In the parent ratings, the average perception of fitness increases steadily with frequency for both genders (from 4.4 to 7.8 for boys and 4.9 to 8.3 for girls). However, gender differences are evident in self-perceptions. While girls’ perceived fitness also increases on average with frequency (from 3.3 to 4.9), there seems to be no clear relationship for boys. With no or little physical activity, they rate their fitness on average considerably better than girls (mean 5.0 for boys vs. 3.3 for girls in the no physical activity group). Compared to the parental assessment, the self-assessment is generally worse, except for the assessment of the boys in the group without physical activity (Figure 8).

The heatmaps revealed that 33% of male patients were tied to ball sports, while 32% of all male patients did not participate in sports (Figure 9). Among female patients, 32% were tied to aesthetic sports, while 24% of all female patients did not participate in sports (Figure 10).

### 3.3 Feasibility

The return of the self-report SPOQ was 100% due to the treatment obligation for inpatients. The individuals needed between five and ten minutes for form completion. The parental-version was an addition to the registration form. The first section was missing 35% of the information, the second section 33%, the third section 54%, the fourth section 37%, and the last section was missing only 6%. We concluded that after completing the 12-page registration form, parents most likely did not want to engage in the specific topic of physical activity. The fewest missing items were in the last section, as this was probably the quickest to answer and, as the last question, had a higher visual focus.

## 4 Discussion

In our study, we capture an overview of the athletic behavior of mentally ill children and adolescents, subjectively assessed physical fitness, and perceived psychological effects of physical activity. One out of 3 patients reported not being physically active at all. Self-reported physical fitness in general declined with age and was perceived to be worst by patients with P/AD and DEPR. Patients with ED reported in particular high frequencies of physical activity and reported to use physical activity as an important resource to cope with their daily lives. The presence of a trainer was generally not considered to be unimportant, except for ADHD patients.

In line with a survey of a mentally healthy sample (118 girls and 127 boys) aesthetic sports for girls and sports games for boys were mentioned as preferred physical activities (36). In our sample 41% of all physically active female patients indicated aesthetic sports, and 55% of all physically active male patients said ball and racket sports were their first sport (Figures 9, 10). Swimming, which was cited in the study of Frömel et al. (36) was cited only occasionally as a preferred sport. Therefore, non-clinical samples may be also usefull to determine which kind of sports may be preferential to support in a clinical sample.

This may also be considered in developing resource-based sports therapy programs. But in particular, the knowledge of sports preference will be helpful to find an optimal sports fit in an expected third of patients who have not been physically active so far.

As expected clinically, individuals with P/AD and DEPR reported also in studies higher scores on negative self-concept as compared with the norm (37–39). This effect could be also seen in our study concerning physical activity (Figure 6).

With respect to gender, self-report (see also Figure 8) showed lower subjectively perceived physical fitness in girls regardless of frequency. There are several possible explanations for this finding. One line of explanation is the experience of negative feedback of peers during sport activity. This explanation is supported the survey of Slater & Tiggemann (40), in which 332 girls and 382 boys aged 12–16 were assessed. Both boys and girls reported being teased and discriminated in sports by same-sex peers. But only girls reported additionally being teased by opposite-sex peers (40). In sum, girl had a higher level of negative experience practicing sports, which can be drawn as one explanation of our finding on gender differences in sport self-image.

In consequence for practical issues regarding improvement of self-efficacy expectations and self-perception of personal strength, targeted interventions should be considered. As in most targeted interventions, this should be started in early ages, where our study in line with others found a higher level of willingness to practice sports. This may be particularly important if sports therapy is used for targeted intervention with patients suffering from P/AD and DEPR.

Since teasing has to be considered as an substantial negative factor for children and adolescents, which let them drop out of sports activity, mixed-gender groups should address non-teasing issues therapeutically right from the beginning.

It has to be highlighted that the subjectively perceived physical fitness follows different dynamics in girls and boys, which is very much in line with the average onset of puberty. While girls in our sample reported lower levels of physical fitness following the age range of 12–13 years, boys had a similar change of scores two years later (14–15 years of age).

Many adult patients with ED use physical activity to regulate negative affect and emotion as a coping strategy and develop a sports addiction (41–43). This deliberate use of physical activity as a coping strategy has been found in children and adolescents with ED (Figure 5) as well. It is highly recommended to pick up their habits and carry out sports therapy in addition to psychotherapy. The aim of combining sports therapy with psychotherapy must be to counter the risk of the development of a sports addiction. Monitored early sports activity can be handled much more effective by the therapist as oppose to self-administered sports without therapeutic framing, which may drive patients into sports addiction more easily.

As was reported in the literature, there was a strong proneness in patients with ED for physical activities related to weight control, such as aesthetic sports and athletics (55 percent, Figure 10) (21, 22). Translating this finding into therapeutic practice, besides starting sports therapy at a young age, the focus should be always set on fun and team-orientation. Sports with a high levels of self-control and weight management should be avoided or at least considered with caution.

Our data indicate that ADHD patients are more likely to value guidance from a trainer compared to other patients in the child and adolescent psychiatric context (Figure 4). They typically find it difficult to structure their actions due to their illness and need this from the outside (44, 45). While parents may lack the educational competence to be supportive in this aspect, children and adolescents receive clear structure, control, and action organization from professional coaches. As a result, they could prove to be more effective in achieving their goals in sports. To conclude, improving the relationship of children and adolescents should be a major focus of sports therapy treatment for children and adolescents with ADHD and may lead to symptom reduction.

### 4.1 Limitations

This study has several limitations: The study design is exploratory in nature. However, this is feasible for developing new instruments in new fields. Additionally, the SPOQ was cross-validated with the Strengths and Difficulties Questionnaire (SDQ) (46) and the EMI-2 (27), but not with other sport-specific instruments. Further, we did not formally test the questionnaire for reliability, which should be addressed in additional studies.

Inpatients and outpatients were not equally distributed between self-rating and parent rating, which limits the interpretation of the data since outpatients usually have lower scores on psychopathology as compared with inpatients. Nevertheless, the observed trend seem to be quite robust against the above mentioned confounding. Thus, the questionnaire evaluated in a population of 313 patients serves to generate hypotheses in an area with highly unmet research needs.

### 4.2 Implications and future directions

Only a few studies examine the athletic behavior of children and adolescents with mental disorders so far. A study with 1.2 million young adults showed that not exercising leads to a higher mental health burden (4). Our results revealed deficits in the frequency of physical activity per week. Almost 1 in 3 patients currently is not physically active (Figures 9, 10). Achieving an appropriate physical activity in children and adolescents should therefore be the primary task of sports therapy. The SPOQ is freely available for other institutions. The next step should be an efficacy study measuring the effects of a sports therapy program and testing hypotheses generated here.

## Data Availability

The datasets presented in this article are not readily available because the data cannot be made available to the public because of the confidentiality of the patient data. Requests to access the datasets should be directed to the data cannot be made available to the public because of the confidentiality of the patient data.
